# Supplementary material for: A Common Polymorphism in RNASE6 Impacts Its Antimicrobial Activity toward Uropathogenic Escherichia coli
Source: Int J Mol Sci. 2024 Jan 3;25(1):604. doi: 10.3390/ijms25010604 (PMC10779065; doi:10.3390/ijms25010604)
Supplement: Supplementary file 1 [file ijms-25-00604-s001.zip › ijms-2733095-Supplementary.pdf]

Supplementary Figures

A Common Polymorphism in *RNASE6* Impacts its Antimicrobial Activity Toward Uropathogenic *Escherichia coli*

Raul Anguita[1+], Guillem Prats-Ejarque[1+], Mohammed Moussaoui[1], Brian Becknell[2*], Ester Boix[1*]

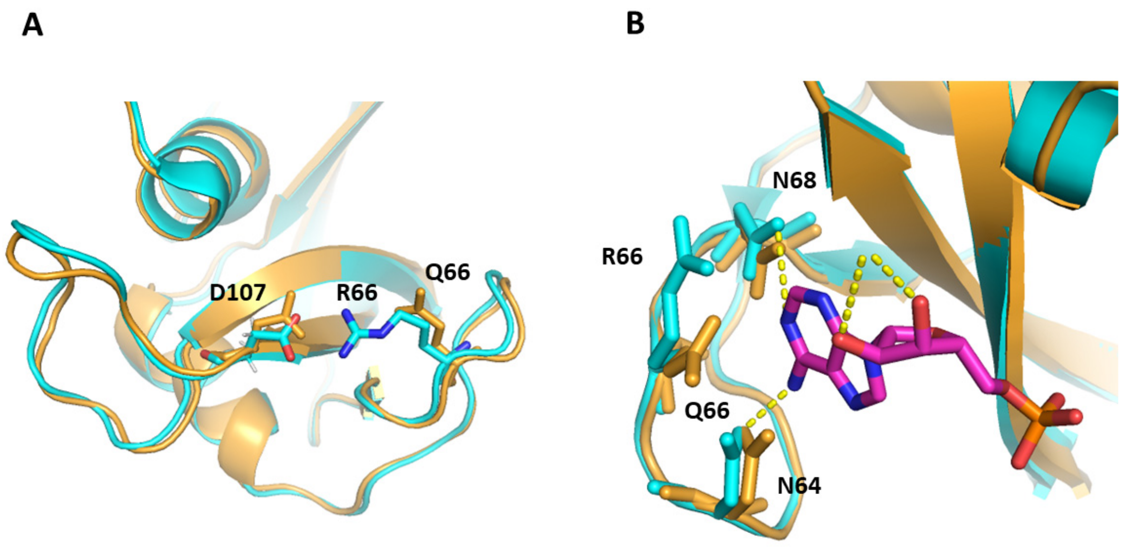

Figure S1. **A)** Comparison of the local 3D structure of RNase 6-R66 and Q66 variants. RNase 6-R66 is drawn in cyan based on the 3D crystal structure (PD ID:5OAB) and RNase 6-Q66 is shown in orange based on the predicted 3D structure by the *AlphaFold2* server. **B)** Comparison of the predicted 3D RNase 6-Q66 structure superimposed onto the RNase 6-R66-AMP complex (PDB ID: 6MV7). Hydrogen bonds to the AMP nucleotide (in magenta) are shown in yellow dashed lines. Key residues at the protein loop interacting with the adenine base are labelled. Picture is drawn with *Pymol* ™ 2.5.2.

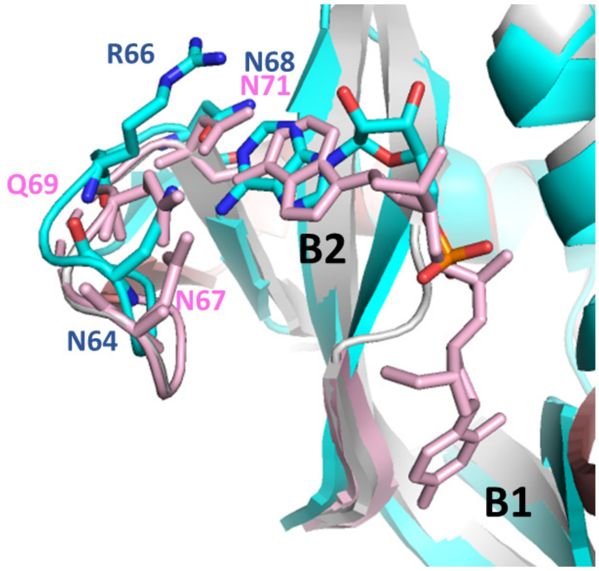

Figure S2. Comparison of the 3D structure of RNase 6-R66 -AMP complex (PDB ID: 6MV7) superimposed onto the RNase A- d(CpA) complex (PDB ID:7RPG). RNase 6 structure is shown in cyan and RNase A in light pink. Key residues at the protein loop interacting with the adenine base are labelled. The RNase main (B1) and secondary base (B2) sites are indicated in black. Picture is drawn with *Pymol* TM 2.5.2.

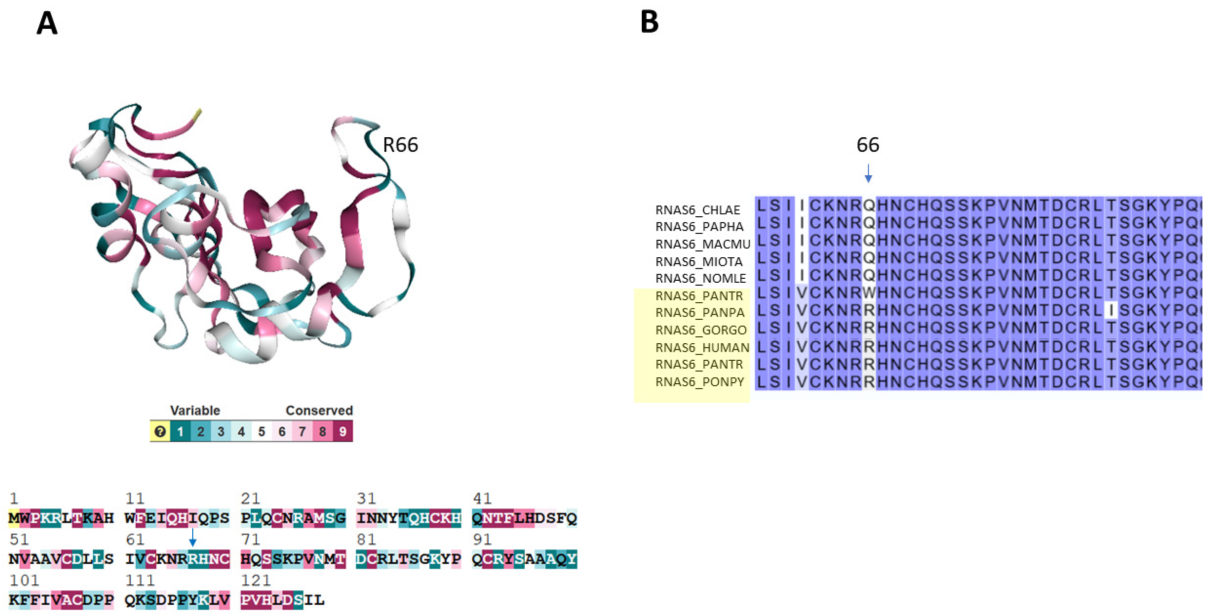

Figure S3. **A)** Identification of conserved and variable residues in the RNase 6 primary sequence based on the analysis of the closest 300 homologues within the RNase A superfamily. Analysis was performed using the *Consurf Server*. Variable and conserved residues are colored based on RNase 6 3D Structure (PD ID: 5OAB) drawn in ribbon. The location of R66 is indicated in the primary sequence, showing its assignment to the highest variability color code.

B) Sequence alignment of RNase 6 homologues showing the amino acid variability at position 66, indicated by an arrow. Hominid species are highlighted with a light yellow box. PANTR corresponds to *Pan troglodites* (chimpazee), PANPA to *Pan paniscus* (pygmy chimpanzee), GORGO to *Gorilla gorilla*, PONPY to *Pongo pygmaeus* (orangutan), NOMLE to *Nomascus leucogenys* (northern white-cheeked gibbon), MIOTA to *Miopithecus talapoin* (southern talapoin), MACMU to *Macaca mulatta* (rhesus macaque), PAPHA to *Papio hamadryas* (hamadryas baboon) and CHLAE to *Chlorocebus aethiops* (grivet). The sequence alignment was obtained using the *BLASTp* suite.
